# Trends of height-for-age Z-scores according to age among Brazilian children under 5 years old from 2006 to 2019

**DOI:** 10.1590/0102-311XEN087222

**Published:** 2023-08-28

**Authors:** Inês Rugani Ribeiro de Castro, Dayana Rodrigues Farias, Talita Lelis Berti, Pedro Gomes Andrade, Luiz Antonio dos Anjos, Nadya Helena Alves-Santos, Elisa Maria de Aquino Lacerda, Maiara Brusco de Freitas, Gilberto Kac, Brazilian Consortium on Child Nutrition

**Affiliations:** 1 Instituto de Nutrição, Universidade do Estado do Rio de Janeiro, Rio de Janeiro, Brasil.; 2 Instituto de Nutrição Josué de Castro, Universidade Federal do Rio de Janeiro, Rio de Janeiro, Brasil.; 3 Departamento de Nutrição Social, Universidade Federal Fluminense, Niterói, Brasil.; 4 Instituto de Estudos em Saúde e Biológicas, Universidade Federal do Sul e Sudeste do Pará, Marabá, Brasil.

**Keywords:** Growth, Body Height, Child Nutrition, Nutritional Status, Nutritional Surveys, Crescimento, Estatura, Nutrição da Criança, Estado Nutricional, Inquéritos Nutricionais, Crecimiento, Estatura, Nutrición del Niño, Estado Nutricional, Encuestas Nutricionales

## Abstract

This study compared the distribution of stunting and height-for-age (HAZ) Z-scores among age groups in data from the *Brazilian National Survey on Demography and Health of Women and Children* (PNDS 2006) and the *Brazilian National Survey on Child Nutrition* (ENANI-2019). The final sample comprised 4,408 and 14,553 children < 59 months of age in the PNDS 2006 and ENANI-2019, respectively. Children with HAZ scores < -2 according to the World Health Organization (WHO) growth standard were classified as stunted. Prevalence, 95% confidence intervals (95%CI), means, and standard deviations were estimated for Brazil and according to age. The distribution of HAZ scores at each age (in months) was estimated using the *svysmooth* function of the R survey package. Analyses considered the complex sampling design of the studies. Statistical differences were determined by analyzing the 95%CI of the overlap of point estimates. From 2006 to 2019, the prevalence of stunting for children < 12 months of age increased from 4.7% to 9%. As expected, the smoothed curves showed a higher mean HAZ score for children < 24 months of age in 2006 than in 2019 with no overlap of 95%CI among children aged 6-12 months. For children ≥ 24 months of age, we observed a higher mean HAZ score in 2019. Although the prevalence of stunting among children < 59 months of age was similar between 2006 and 2019, mean HAZ scores among children ≥ 24 months of age increased, whereas the mean HAZ score among children < 24 months of age decreased. Considering the deterioration in living conditions and the potential impact of the COVID-19 pandemic, we expect a greater prevalence of stunting in Brazil in the near future.

## Abstract (pt)

Este estudo comparou a distribuição dos escores Z de estatura (ZAI) e déficit de estatura por faixas etárias nos dados da *Pesquisa Nacional de Demografia e Saúde da Criança e da Mulher* (PNDS 2006) e da *Pesquisa Nacional de Nutrição Infantil* (ENANI-2019). Nossa amostra final foi composta por 4.408 e 14.553 crianças < 59 meses de idade da PNDS 2006 e ENANI-2019, respectivamente. Crianças com escores HAZ < -2 de acordo com o padrão de crescimento da Organização Mundial da Saúde (OMS) foram classificadas como tendo déficit de estatura. Prevalências, intervalos de 95% de confiança (IC95%), médias e desvios padrão foram estimados para o Brasil e de acordo com a idade. A distribuição dos HAZ em cada idade (em meses) foi estimada usando a função *svysmooth* do pacote R. Nossas análises consideraram o desenho amostral complexo dos estudos. Diferenças estatísticas foram determinadas pela análise da sobreposição pontual dos IC95%. Entre 2006 e 2019, a prevalência de déficit de estatura para crianças < 12 meses de idade aumentou de 4,7% para 9%. Como esperado, as curvas suavizadas revelaram um HAZ médio maior para crianças < 24 meses de idade em 2006 do que em 2019, sem sobreposição de IC95% entre crianças de 6-12 meses. Para crianças ≥ 24 meses de idade, observamos um HAZ médio maior em 2019. Embora a prevalência de déficit de estatura entre crianças < 59 meses de idade tenha sido semelhante entre 2006 e 2019, observamos um aumento no HAZ médio entre crianças ≥ 24 meses de idade e uma diminuição no HAZ médio entre crianças < 24 meses de idade. Considerando a deterioração das condições de vida e o potencial impacto da pandemia de COVID-19, espera-se uma maior prevalência de déficit de estatura no Brasil no futuro próximo.

## Abstract (es)

Este estudio comparó la distribución de las puntuaciones Z de talla (ZTE) y el déficit de estatura por grupos de edad en los datos de la *Encuesta Nacional de Demografía y Salud del Niño y de la Mujer* (PNDS 2006) y la *Encuesta Nacional de Nutrición Infantil* (ENANI-2019). Nuestra muestra final consistió en 4.408 y 14.553 niños < 59 meses de edad de PNDS 2006 y ENANI-2019, respectivamente. Los niños con puntuaciones HAZ < -2 según el patrón de crecimiento de la Organización Mundial de la Salud (OMS) se clasificaron como con déficit de talla para edad. Las prevalencias, los intervalos de 95% de confianza (IC95 %), las medias y las desviaciones estándar se estimaron para Brasil y según la edad. La distribución de HAZ para cada edad (en meses) se estimó utilizando la función *svysmooth* del paquete R. Nuestros análisis tuvieron en cuenta el complejo diseño de muestra de los estudios. Las diferencias estadísticas se determinaron mediante el análisis de la superposición puntual de los IC95 %. Entre 2006 y 2019, la prevalencia del déficit de talla para edad en niños < 12 meses de edad aumentó del 4,7 % al 9%. Como se esperaba, las curvas suavizadas revelaron un HAZ promedio mayor para los niños < 24 meses de edad en 2006 que en 2019, sin una superposición del IC95 % entre los niños de 6-12 meses. Para los niños ≥ 24 meses de edad, observamos un HAZ promedio mayor en 2019. Aunque la prevalencia del déficit de talla para edad entre los niños < 59 meses de edad fue similar entre 2006 y 2019, observamos un aumento en el HAZ promedio entre los niños ≥ 24 meses de edad y una disminución en el HAZ promedio entre los niños < 24 meses de edad. Teniendo en cuenta el deterioro de las condiciones de vida y el impacto potencial de la pandemia de COVID-19, se espera una mayor prevalencia de déficit de talla para edad en Brasil en un futuro cercano.

## Introduction

Comparing the results of the *Brazilian National Survey on Demography and Health of Women and Children* (PNDS 2006) ^1^ with those of the *Brazilian National Survey on Child Nutritio*n (ENANI-2019) ^2^ shows a transition pattern in which different nutritional problems coexist in the same population. From 2006 to 2019 the prevalence of anemia (20.5% vs. 10.1%) and vitamin A deficiency (17.2% vs. 6%) decreased among children 6-59 months of age; excess weight increased (6% vs. 10.1%), stunting rates remained similar (7.3% in 2006 and 7% in 2019) among children 0-59 months of age; and the prevalence of exclusive breastfeeding increased in children < 6 months of age (37.1% vs. 45.8%).

This study hypothesized that the stability in stunting point estimates among children < 59 months of age do not reveal trends in linear growth in specific age groups. This study aimed to explore and compare stunting prevalence and height-for-age Z (HAZ) score distribution between data from the PNDS 2006 and ENANI-2019 according to children’s age.

## Methods

This descriptive study analyzed microdata from the PNDS 2006 (n = 4,817) and ENANI-2019 (n = 14,558), population-based household surveys conducted with complex probability sampling, conglomeration, and stratification to ensure macroregion representativeness of Brazilian children < 59 months of age ^1^ ^,^ ^3^. Data from the 2008-2009 *Brazilian Household Budget Survey* (POF), the only national survey conducted from 2006 to 2019 that collected anthropometric measurements of children < 59 months of age were not included in this study because these data had a high percentage of biologically implausible HAZ scores (2.1%), indicating insufficient precision in height measurements of children < 59 months of age ^4^.

In the PNDS 2006, children’s height was measured twice using a portable stadiometer for children ≥ 24 months of age and an infantometer for those < 24 months of age. The portable devices for height measurement (i.e., the stadiometer and infantometer) were developed by the team at the Laboratory of Nutritional Assessment of Populations (LANPOP) at the School of Public Health, University of São Paulo ^1^. The first height measurement was used to obtain the Z-score based on the World Health Organization (WHO) reference curves ^5^. Children missing values for the first measurement of height (n = 383) or measuring method (standing or lying down) (n = 2) and those with implausible HAZ scores (n = 24) (Z < -6 or Z > 6 according to the WHO growth standard ^5^) were excluded (total loss: 8.45%). The final sample comprised 4,408 children. The calculation of the child’s age (in days) was performed based on the child’s date of birth and the day the anthropometric measurements were collected.

In the ENANI-2019, children’s height was measured twice using a portable stadiometer for children ≥ 24 months of age and an infantometer for children < 24 months of age. Measurements were performed using equipment specifically acquired for the survey (SECA; https://www.seca.com/). The first height measurement was inputted when necessary (i.e., for missing or implausible data) and used to classify nutritional status ^6^. In total, five children who had some physical disability or other condition that prohibited height measurements were excluded (0.03%). The final sample comprised 14,553 children. The first height measurement was used to obtain the Z-score based on WHO reference curves ^5^. For children who were born < 37 weeks of pregnancy and who, at the time of the study, were aged from 189-454 days since conception (calculated by summing gestational age at birth and age in postnatal days), the postnatal growth curves of the INTERGROWTH-21^st^ Project ^7^ were used.

Stunting prevalence (HAZ < -2) and their respective 95% confidence intervals (95%CI) were estimated for Brazil and according to the following age groups: 0-11, 12-23, 24-35, 36-47, and 48-59 months, whereas means, confidence intervals, and standard deviations of HAZ scores were estimated for Brazil and the following age groups: < 24 and ≥ 24 months and 0-11, 12-23, 24-35, 36-47, and 48-59 months. Mean HAZ scores were stratified and analyzed into 24 months of age for two reasons: (a) children aged < 24 months show a faster growth rate and greater vulnerability to morbidities than older children ^8^ ^,^ ^9^ and (b) children in this age group in the ENANI-2019 were born in a more adverse sociopolitical and economic context than those ≥ 24 months of age ^10^. The distribution of HAZ scores according to age (in months) in each study was evaluated using the *svysmooth* function of the survey package ^11^. The Loess method was used considering the sampling design ^1^ ^,^ ^3^. Analyses were performed in the R (http://www.r-project.org) programming language. Significant differences were examined by analyzing the overlap of the 95%CI of point estimates. A comparison of confidence intervals was adopted instead of a formal statistical test for differences because the databases of each survey were analyzed separately since they had different sampling weights and sampling strategies ^1^ ^,^ ^3^. These methodological differences (in addition to the temporal distance between the studies) impact the application of advanced statistical tests to compare estimates between surveys and demand a model that considers confounding factors, going beyond the descriptive objective of this study.

## Results

**Figure 1:**
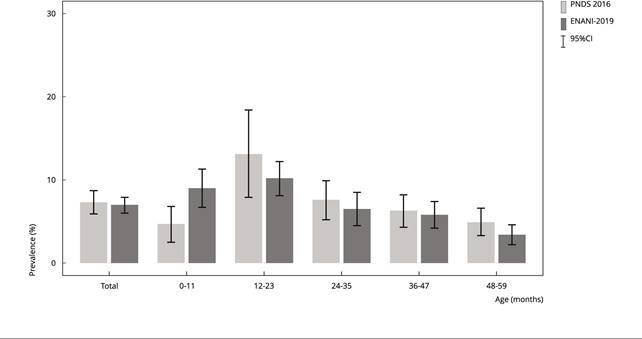
Prevalence of low height for age (Z < -2) in children < 5 years old for Brazil and according to age group (in months). Brazil, 2006 and 2019.

The prevalence of stunting in children < 59 months of age in Brazil was similar in 2006 and 2019. However, stratification according to age groups suggested differences in the distribution of stunting, despite the overlap of 95%CI. We found a stunting prevalence of 8.8% (95%CI: 5.9; 11.7) and 9.6% (95%CI: 8.0; 11.1) in the PNDS 2006 and ENANI-2019 among children < 24 months of age, respectively. Children ≥ 24 months of age showed a 6.3% (95%CI: 5.1; 7.4) and 5.2% (95%CI: 4.2; 6.3) prevalence, respectively (data not shown). Unlike other age groups, the prevalence of stunting increased among children 0-11 months of age, from 4.7% in 2006 to 9% in 2019, with overlapping 95%CI (Figure 1).

95%CI: 95% confidence interval; ENANI-2019: *Brazilian National Survey on Child Nutrition*; PNDS 2006: *Brazilian National Survey on Demography and Health of Women and Children*.

Mean HAZ scores in the PNDS 2006 and ENANI-2019 statistically differed for children ≥ 24 months of age [-0.43 (95%CI: -0.51; -0.36) vs. -0.27 (95%CI: -0.32; -0.21)] and were borderline for children < 24 months of age [-0.15 (95%CI: -0.28; -0.20) vs. -0.35 (95%CI: -0.42; -0.28)]. We observed a decrease in mean HAZ scores among children < 12 and 12-23 months of age. We found an increase in the mean HAZ score for the other age groups over time. Both situations showed overlapping 95%CI (Table 1).

**Table 1:** Mean, 95% confidence interval (95%CI), and standard deviation (SD) of the height for age Z-score of children < 5 years old according to age group (months). Brazil, 2006 and 2019.

Age (months)	PNDS 2006			ENANI-2019		
	Mean	95%CI	SD	Mean	95%CI	SD
Total	-0.32	-0.40; -0.24	1.20	-0.30	-0.35; -0.26	1.25
0-23	-0.15	-0.28; -0.02	1.31	-0.35	-0.42; -0.28	1.39
24-59	-0.43	-0.51; -0.36	1.11	-0.27	-0.32; -0.21	1.15
0-11	0.00	-0.17; 0.18	1.20	-0.20	-0.29; -0.10	1.41
12-23	-0.31	-0.50; -0.12	1.39	-0.51	-0.60; -0.42	1.35
24-35	-0.50	-0.62; -0.39	1.11	-0.38	-0.47; -0.28	1.21
36-47	-0.45	-0.57; -0.33	1.13	-0.28	-0.36; -0.20	1.14
48-59	-0.34	-0.48; -0.20	1.08	-0.15	-0.24; -0.05	1.09

ENANI-2019: *Brazilian National Survey on Child Nutrition*; PNDS 2006: *Brazilian National Survey on Demography and Health of Women and Children*.

**Figure 2:**
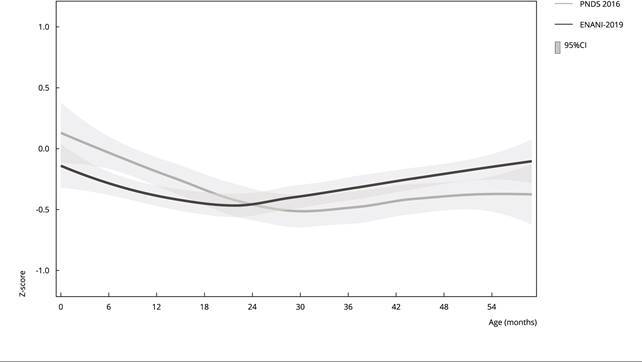
Smoothed Z-score curves of the height-for-age index in children < 5 years old. Brazil, 2006 and 2019.

The smoothed distribution curves of mean HAZ scores according to age group also showed differences between surveys. In the PNDS 2006, children < 24 months of age had higher mean HAZ scores than those in the ENANI-2019. We found a reverse pattern among children ≥ 24 months of age: children surveyed in the ENANI-2019 had higher mean HAZ scores than those in the PNDS 2006 (Figure 2). The 95%CI of both survey estimates only failed to overlap with each other for children between 6 and 12 months of age.

95%CI: 95% confidence interval; ENANI-2019: *Brazilian National Survey on Child Nutrition*; PNDS 2006: *Brazilian National Survey on Demography and Health of Women and Children*. Note: smoothed curve estimated by the Loess method.

## Discussion

This study showed that the stability in point estimates of stunting prevalence between 2006 and 2019 among children < 59 months of age concealed the temporal trend of linear growth in different age groups. The changes in patterns we observed among children ≥ 24 months of age suggest that older children from the ENANI-2019 grew up in better living conditions than those from the PNDS 2006, which may result from a virtuous cycle of economic, health, and food security policies ^12^ ^,^ ^13^. In contrast, the changes observed among children < 24 months of age (especially < 12-month-old ones), who were born after 2017, may reflect exposure to adverse socioeconomic and health conditions.

Notably, as a limitation of the study, the classification of nutritional status in children < 24 months of age was only partially comparable between the two studies because the PNDS 2006 ignored children’s preterm conditions, which could have led it to overestimate stunting prevalence. However, the impact of preterm birth is less critical to length measurements and more relevant for other anthropometric measurements, such as weight and head circumference ^7^. Our analyses of ENANI-2019 data confirmed this conclusion by INTERGROWTH-21^st^ Project researchers ^7^ since we compared results both correcting and not correcting for preterm birth: results showed very similar stunting prevalence rates. Therefore, it is safe to assume a negligible role of this limitation on such estimates.

National data showed an improvement in social inequality, a decrease in poverty, an improvement in access to education, and economic growth in the early 2000s ^12^, with a reduction in the prevalence of stunting from 13.4% to approximately 7% from 1996 to 2006 ^13^. However, since 2015, social inequality, poverty, and food insecurity has increased in Brazil ^12^ ^,^ ^14^. This stems from the economic recession in this period, followed by fiscal austerity measures and the dismantling of rights guarantee policies ^14^. Such an adverse context may have affected the linear growth of children given that it is a cumulative measure sensitive to living conditions. Early life, particularly its first 24 months, undergoes rapid growth and development that can be compromised by adverse living conditions and lead to increased morbidities and decreased growth rates ^8^ ^,^ ^9^. Therefore, younger children’s (< 24 months of age) growth may have suffered more intense consequences than that of older children (≥ 24 months of age).

When compared to the PNDS 2006 ^1^, ENANI-2019 also showed worsened thinness indicators (body mass index - BMI-for-age Z-scores < -2 according to growth standards; increasing from 1.7% in 2006 to 3% in 2019) and underweight for age (weight-for-age Z-scores < -2 according to growth standards; increasing from 1.9% in 2006 to 2.9% in 2019), despite overlapping confidence intervals, for children < 59 months of age ^15^. Weight measures are more sensitive to short-term changes than height measures and may indicate a shift in the more recent nutritional context.

Assuming that the profile of linear growth we observed in the ENANI-2019 among children < 24 months of age remains thus, we can expect an increase in the prevalence of stunting among children < 59 months of age in the near future. The effects of the COVID-19 pandemic may worsen this context ^16^ ^,^ ^17^.
